# Enhanced production of thermostable amidase from *Geobacillus subterraneus* RL-2a MTCC 11502 via optimization of physicochemical parameters using Taguchi DOE methodology

**DOI:** 10.1007/s13205-016-0390-1

**Published:** 2016-02-15

**Authors:** Praveen Kumar Mehta, Shashi Kant Bhatia, Ravi Kant Bhatia, Tek Chand Bhalla

**Affiliations:** Department of Biotechnology, Himachal Pradesh University, Simla, 171 005 India

**Keywords:** *Geobacillus subterraneus*, Optimization, Orthogonal array, Production, Amidase

## Abstract

The specific effect of chemical and physical factors on amidase production from *Geobacillus subterraneus* RL-2a was investigated using design of experiments (DOE) methodology. The one-factor-at-a-time (OFAT) method was used to study the effects of carbon and nitrogen sources on amidase production. Subsequently, optimal levels of physical parameters and key media components, namely temperature, pH, sucrose, K_2_HPO_4_, NaCl, yeast, CaCl_2_·2H_2_O and MgSO_4_·7H_2_O, were determined using the Taguchi orthogonal array (OA) experimental design (DOE) methodology. Taguchi method based on three levels with a OA layout of L18 (2^1^ × 3^7^) with eight most influential factors on amidase synthesis for the proposed experimental design. Analysis of variance was performed on the obtained results and optimum condition suggested by statistical calculations was tested in a verification test. An increase of 169.56 % in amidase production compared to the unoptimized conditions was observed and the conversion of isonicotinamide was significantly improved after performing optimization techniques, including OFAT and Taguchi method. The result indicated that Taguchi method was effective in optimizing the culture conditions of amidase production.

**Electronic supplementary material:**

The online version of this article (doi:10.1007/s13205-016-0390-1) contains supplementary material, which is available to authorized users.

## Introduction

Amidase, an amide hydrolyzing enzyme, is a member of nitrilase super family and has a great potential for the transformation of amides to corresponding acids and ammonia. Amidase activity has been described in many members of bacterial kingdom including *Arthrobacter* (Fournand and Arnand 2001), *Bacillus* (Kim and Oriel 2000), *Geobacillus* (Makhongela et al. 2007), *Microbacterium* (Doran et al. 2005), *Pseudomonas* (Egorova et al. 2004) and *Rhodococcus* (Trott et al. 2002).

Amidases are one of the most extensively used amide-hydrolysing enzymes in industry due to their enantio/stereoselective properties and capacity for the large-scale production of optically pure organic acids such as p-aminobenzoic acid, nicotinic acid and acrylic acid (Banerjee et al. 2002; Hirrlinger et al. 1996; Wang et al. 2005).

Isonicotinic acid or pyridine-4-carboxylic acid is an important pyridine derivative which is mainly used for the synthesis of isoniazid (an antituberculastic drug), inabenfide (a plant growth regulator), terefenadine (an antihistamine) and nialamide (an antidepressant) (Yadav et al. 2005; Scriven et al. 1998). The chemical processes that are used for the manufacturing of isonicotinic acid are hazardous, energy-demanding and expensive (Yadav et al. 2005). The isonicotinamide can be converted to isonicotinic acid by amidase enzyme as per reaction scheme given below:
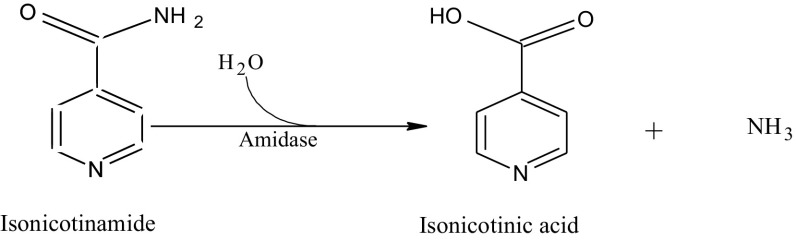
To date, however, there are very scanty reports on isonicotinamide hydrolysis to produce isonicotinic acid by thermophilic bacterium (Egorova et al. 2004; d’Abusco et al. 2001).

In the conventional one-factor-at-a-time method for optimizing fermentation medium conditions (i.e. nutrients, temperature, pH, etc.), one independent variable is changed while all others are held at definite levels. This one-dimensional evaluation is tedious and time-consuming and requires more experiments and cannot provide information about the mutual interactions of the parameters.

On the other extreme, there are full-factorial experimental designs that consider all variables and their interactions at once. But this dedicates huge numbers of trials, which have to be done, most probably beyond the time and budget capabilities. As a solution, fractional factorial experimental designs, including Placket–Burman design, orthogonal array and response surface methodology (RSM) designs have been introduced, reducing the number of the tests while giving reliable results (Greasham and Inamine 1986; Strobel and Sulivan 1999; Xu et al. 2003).

The concept of design of experiments (DOE) makes the optimization easy, helps in building the models, evaluating the effect of several factors, achieving the optimum conditions for desirable responses and also reduced the number of experiments to large extent (Montgomery 2005). This methodology is applied in many engineering areas and is extensively used to design robust products and processes for industry. Its application in the biotechnology field, in particular in fermentation processes, has also proven to be useful (Rao et al. 2008).

The purpose of this study was to optimize culture condition to improve amidase production by *G. subterraneus* RL-2a using one-factor-at-a-time and a statistics-based orthogonal array designs. At first, the effects of different medium, carbon and nitrogen sources on amidase production were investigated by one-factor-at-a-time. Then, the concentration of medium components was optimized using taguchi method as a fractional factorial design.

## Materials and methods

### Chemicals

Isonicotinamide and isonicotinic acid were purchased from Alfa Aesar, A Johnson Matthey Company. All the culture media ingredients were procured from Hi Media (Mumbai, India). For high-pressure liquid chromatography, HPLC grade solvents were purchased from Merck, India.

### Microorganism and growth conditions

An Amidase producing potent bacterium *G. subterraneus* RL-2a (accession no. MTCC 11502) was used in the present experiment. It was isolated from Manikaran thermal spring (Himachal Pradesh, India) and deposited in the Microbial Type Culture Collection and Gene Bank, Institute of Microbial Technology, Chandigarh, India, and was maintained on a nutrient agar medium (Mehta et al. 2013).

Preculture was prepared by transferring a single colony of *Geobacillus subterraneus* RL-2a grown over nutrient agar for 48 h at 50 °C to 50 ml medium containing (0.5 %) peptone, (0.15 %) beef extract, (0.15 %) yeast extract and (0.5 %) NaCl (pH 7.0) in a 250-ml Erlenmeyer flask and incubated for 17 h, 150 rpm in an incubator shaker at 50 °C. The preculture 5 % (v/v) in the late exponential phase was inoculated into 50 ml of minimal salt medium (MSM) containing (per litre) 2.5 g Na_2_HPO_4_·12H_2_O, 2.0 g KH_2_PO_4_, 0.03 g FeSO_4_·7H_2_O, 0.5 g MgSO_4_·7H_2_O, 0.06 g CaCl_2_·2H_2_O and 1 % (w/v) glucose as carbon source in 250 ml Erlenmeyer flask and incubated at 50 °C, 150 rpm in an incubator shaker. After specific incubation time, the cells from the culture were harvested by centrifugation at 15,000*g* (4 °C, 15 min) and washed twice with 0.1 M potassium phosphate buffer (pH 7.0) for further use.

### Enzyme assay

The amidohydrolase assay was performed in a reaction mixture (2.0 ml) containing isonicotinamide as substrate in 0.1 M potassium phosphate buffer (pH 7.0), and the appropriate amount of resting cells at 70 °C in a water bath shaker. After 20 min of incubation, reaction was stopped with equal volume of 0.1 N HCl. The amount of ammonia released in the reaction mixture was colorimetrically estimated using phenate-hypochlorite method (Fawcett and Scott 1960). One unit (U) of amidase activity was defined as that amount of resting cells (mg dry cell = mg dcw) required to release 1 µmol/min of ammonia by the hydrolysis of amide under assay conditions.

### Determination of bioconversion by HPLC

The amount of isonicotinic acid produced in the reaction mixture was determined using series 200 IC pump (Perkin Elmer) equipped with Inertsil^®^ ODS-3 5 μm (4.6 × 150 mm) column (GL Sciences, Japan) and 785A Programmable Absorbance Detector (Applied Biosystem). Chromatogram was monitored at 230 nm using mobile phase 0.01 M KH_2_PO_4_/H_3_PO_4_ buffer (pH 2.8)/acetonitrile (4:1, v/v) at a flow rate of 1.0 ml/min using NetWin Software (Netel Chromatographs, India). The calibration curves for isonicotinamide (0.1–1.0 mM) and isonicotinic acid (0.1–1.0 mM) were prepared.

### Optimization of media for amidase production

The optimization of medium constituents for amidase production by *G. subterraneus* RL-2a was carried. Initially, selection of medium components including carbon sources and nitrogen sources was investigated through the traditional ‘one-variable-at-a-time’ approach (OVAT), and then the concentration of each optimized constituent was determined by orthogonal matrix method.

### Growth medium for amidase production

The *G. subterraneus* RL-2a was grown in 12 different media at 50 °C, out of which ten were already reported in literature and the rest were self-formulated. The initial pH for all media was adjusted to 7.0. The composition of different media is summarized in Table 1. The amidase activity of the cells grown in various media was assayed as mentioned above.

**Table 1:** Different media used for the amidase production by *G. subterraneus* RL-2a

Medium code	Composition (g/l)		Growth (mg dcw/ml)	Activity (U/mg dcw)	Reference
M1	Peptone	5.0 g	0.12	0.10	Nutrient broth
	Beef extract	3.0 g			
M2	Peptone	12.5 g	0.04	0.07	Piotraschke et al. (1994)
	Beef extract	3.0 g			
	Yeast extract	5.0 g			
	NaCl	5.0 g			
M3	Glucose	15.0 g	0.05	0.06	Rao et al. (2008)
	Peptone	5.0 g			
	Malt extract	3.0 g			
	Yeast extract	3.0 g			
M4	Glycerol	10.0 g	0.17	0.09	Robas et al. (1993)
	Peptone	5.0 g			
	Malt extract	3.0 g			
	Yeast extract	3.0 g			
M5	Glucose	10.0 g	0.09	0.21	Scriven et al. (1998)
	Na_2_HPO_4_·12H_2_O	2.5 g			
	K_2_HPO_4_	2.0 g			
	MgSO_4_·7H_2_O	0.5 g			
	FeSO_4_·7H_2_O	0.3 g			
	CaCl_2_·2H_2_O	0.6 g			
	Yeast extract	1.0 g			
M6	(NH_4_)_2_HPO_4_	5.0 g	0.09	0.20	APY mineral salt medium
	Peptone	5.0 g			
	Yeast extract	3.0 g			
	K_2_HPO_4_	5.0 g			
	MgSO_4_·7H_2_O	0.2 g			
	FeSO_4_·7H_2_O	0.02 g			
M7	Glycerol	10.0 g	0.19	0.09	Strobel and Sulivan (1999)
	K_2_HPO_4_	0.5 g			
	KH_2_PO_4_	0.5 g			
	MgSO_4_·7H_2_O	0.1 g			
	Yeast extract	1.0 g			
	Peptone	5.0 g			
M8	Peptone	20.0 g	0.13	0.06	Trott et al. (2002)
	NaCl	5.0 g			
	Glucose	2.0 g			
	Yeast extract	3.0 g			
	Beef extract	3.0 g			
M9	Tryptone	30.0 g	0.14	0.13	Vaidya et al. (2009)
	Yeast extract	15.0 g			
	NaCl	5.0 g			
	Glucose	2.0 g			
M10	K_2_HPO_4_	2.0 g	0.04	0.10	Venkata Mohan et al. (2007)
	NaCl	1.0 g			
	MgSO_4_	0.01 g			
	FeSO_4_·7H_2_0	0.02 g			
	Biotin	2 × 10^−5^ g			
	Thiamine	0.004 g			
	Inositol	0.002 g			
M11	Peptone	12.5 g	0.11	0.09	Self-formulated
	Yeast extract	3.0 g			
	Beef extract	5.0 g			
	NaCl	5.0 g			
	FeSO_4_·7H_2_0	0.01 g			
M12	Glycerol	10.0 g	0.12	0.23	Self-formulated
	NaCl	2.0 g			
	K_2_HPO_4_	1.0 g			
	MgSO_4_·7H_2_O	0.5 g			
	Beef extract	0.2 g			
	CaCl_2_	0.3 g			

### Effect of carbon sources

Different carbon sources (1 %, w/v) like monosaccharides (e.g. glucose, mannitol, arabinose and galactose), disaccharides (e.g. sucrose, lactose, maltose), polysaccharides (e.g. starch) and some inorganic carbon source (sodium succinate, sodium citrate, sodium acetate) were used to find out the most suitable one for the growth and production of amidase by *G. subterraneus* RL-2a. The best carbon source was utilized for subsequent study.

### Effect of organic nitrogen sources

To investigate the effect of various organic nitrogen sources on the production of amidase by *G. subterraneus* RL-2a, different organic nitrogen sources (0.3 % w/v) (peptone, beef extract, yeast extract, tryptone, urea) were added to the medium. Similarly, the effect of various inorganic nitrogen sources (ammonium nitrate, ammonium sulphate, ammonium chloride, sodium nitrate, di-ammonium sulphate) was supplemented in the culture medium and their influences on enzyme production of amidase by *G. subterraneus* RL-2a were evaluated.

### Taguchi DOE methodology

A standard orthogonal array (OA) L18 (2^1^ × 3^7^) with three levels of factor variation was used in this optimization procedure. All of these factors were assigned with three levels, except incubation temperature which was assigned with two levels. The L and the subscript (18) represent the Latin square and the number of experimental runs, respectively.

In Taguchi’s method, quality is measured by the deviation of a characteristic from its target value and a loss function [*L*(*y*)] is estimated for the deviation as *L*(*y*) = *k* × (*y* − *m*)^2^, where *k* denotes the proportionality constant, *m* represents the target value and *y* is the experimental value obtained for each trail (Mitra 1998). In case of bigger and better quality characteristics, the loss function can be written as *L*(*y*) = *k* × (1/*y* ^2^) and the expected loss function can be represented by

$$E[L(y)] = k \times E(1/y^{2} ) ,$$

where *E*(1/*y* ^2^) can be estimated from n number of samples as

$$\mathop \sum \limits_{i = 1}^{n} (1/y^{2} )/n$$

Taguchi method involves establishment of large number of experimental situations described as OAs to reduce errors and to enhance the efficiency and reproducibility of the laboratory experiments. The designed approach was broadly divided into four phases (with various steps): planning, experimental, analysis and validation (Venkata Mohan et al. 2007). Each phase had a separate objective, interconnected in sequence to achieve the overall optimization process.

### Experimental design

The first phase focused on the composition of the factors to be optimized in the culture medium that has critical effect on the amidase yield. Based on the obtained experimental data from our initial studies, eight factors were selected for the production of amidase by *G. subterraneus* (Table 2).

**Table 2:** Selected culture condition factors and assigned levels

S. No.	Factors	Level-1	Level-2	Level-3
1	pH	6	7	–
2	Temperature (°C)	45	55	65
3	Carbon source (g%, w/v as sucrose)	0.5	1.0	1.5
4	Phosphate source (g%, w/v as K_2_HPO_4_)	0.1	0.25	0.5
5	Nitrogen source (g%, w/v as yeast extract)	0.01	0.02	0.03
6	Na^+^ (g%, w/vas NaCl)	0.25	0.5	0.75
7	Magnesium sulphate (g%, w/v)	0.01	0.025	0.05
8	Calcium Chloride (g%, w/v)	0.025	0.050	0.075

Submerged fermentation was carried out in a 250-ml Erlenmeyer flask containing 50 ml of production media, which was prepared by varying the composition (g%, w/v) of carbon source (sucrose: 0.5, 1.0 and 1.5): phosphate source (K_2_HPO_4_: 0.1, 0.25 and 0.5); nitrogen source (yeast extract: 0.01, 0.02 and 0.03) and metal ion (MgSO_4_·7H_2_O: 0.01, 0.025 and 0.05; NaCl: 0.25, 0.5 and 0.75; CaCl_2_·2H_2_O: 0.025, 0.05 and 0.075); pH of the production media was adjusted to 6.0, and 7.0 and fermentation was performed at different simulated temperatures (45, 55 and 65 °C). The level of amidase activity presented as the mean value of three individual assays.

The second step was to design the matrix experiment and to define the data analysis procedure. The levels of the factors studied and the layout of the L18 Taguchi’s orthogonal array are shown in Table 3. The experimental results were analysed to extract independently the main effects of the factors; the analysis of variance technique was then applied to determine which factors were statistically significant. The controlling factors were identified, with the magnitude of effects qualified and the statistically significant effects determined. Accordingly, identification of the individual influence of each factor, determination of the optimum condition and estimation of performance at the optimum condition were determined. All calculations were performed using Qualitek-4 (Automatic Design and Analysis of Taguchi Experiment) Nutek, Inc. USA. To validate the determined optimized methodology, further submerged fermentation experiments were performed using the obtained optimized culture conditions.

**Table 3:** L18 (2^1^ × 3^7^) orthogonal array of designed experiments

S. No.	1 (pH)	2 (Temp)	3 (Sucrose)	4 (K_2_HPO_4_)	5 (NaCl)	6 (yeast extract)	7 (MgSO_4_)	8 (CaCl_2_)	Amidase^a^ activity (U/mg dcw)
1	1	1	1	1	1	1	1	1	0.14
2	1	1	2	2	2	2	2	2	0.32
3	1	1	3	3	3	3	3	3	0.04
4	1	2	1	1	2	2	3	3	0.07
5	1	2	2	2	3	3	1	1	0.03
6	1	2	3	3	1	1	2	2	0.69
7	1	3	1	2	1	3	2	3	0.06
8	1	3	2	3	2	1	3	1	0.14
9	1	3	3	1	3	2	1	2	0.02
10	2	1	1	3	3	2	2	1	0.11
11	2	1	2	1	1	3	3	2	0.08
12	2	1	3	2	2	1	1	3	0.28
13	2	2	1	2	3	1	3	2	0.37
14	2	2	2	3	1	2	1	3	0.13
15	2	2	3	1	2	3	2	1	0.05
16	2	3	1	3	2	3	1	2	0.05
17	2	3	2	1	3	1	2	3	0.10
18	2	3	3	2	1	2	3	1	0.09

^a^Values indicate mean of duplicate observations

## Results and discussion

### Selection of medium for amidase production

In order to investigate the effect of nutritional media on the growth and production of amidase by the *G. subterraneus* RL-2a, it was grown in twelve media and results are summarized in Table 1. The results suggested that the medium M-12 (pH 7.0) containing (g/l) 10 g glycerol, 2.0 g NaCl, 1.0 g K_2_HPO_4_, 0.5 g MgSO_4_·7H_2_O, 0.3 g CaCl_2_·2H_2_O and 0.2 g beef extract was found to be most suitable for the production of amidase. The maximum amidase activity obtained with this medium was 0.23 U/mg dcw with 0.12 mg/ml of cell mass. The organism grew well in most of the media and maximum yield of cell mass was observed in M7 medium. The next best medium was found to be M-5 exhibiting 0.21 U/mg dcw amidase activity; however, the cell mass obtained was 0.09 mg/ml. *G. subterraneus* RL-2a exhibited good biomass production in M-1, M-4, M-7 and M-9, but production of amidase activity was low (Table 1).

### Effect of carbon source

**Fig. 1:**
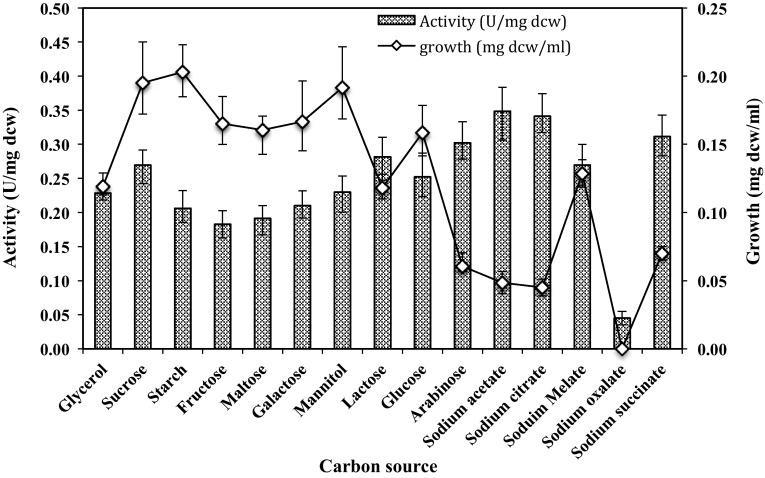
Amidase production by *G. subterraneus* RL-2a using various 5.0 % (w/v) carbon sources

Carbon source has a crucial role in the determination of the growth and metabolic rates of microorganisms. A number of carbon sources [1.0 % (w/v)] were tested to enhance the growth and production of amidase activity of *G. subterraneus* RL-2a (Fig. 1). Inorganic carbon sources such as sodium acetate, sodium citrate, sodium melate and sodium succinate did not support strain growth. Low enzyme activity was obtained with fructose, maltose, galactose, starch and mannitol, although they supported excellent biomass production. Inorganic carbon source such as sodium oxalate did not support amidase production in *G. subterraneus* RL-2a. Amidase activity was found to be much higher when other carbon sources like arabinose, sodium acetate, sodium citrate, sodium succinate and sodium melate were used in the media; however, the latter carbon source at the same time did not yield higher cell mass. The highest amidase activity (0.27 U/mg dcw) and biomass (0.19 mg dcw/ml) were achieved with sucrose as carbon source.

### Effect of nitrogen source

**Fig. 2:**
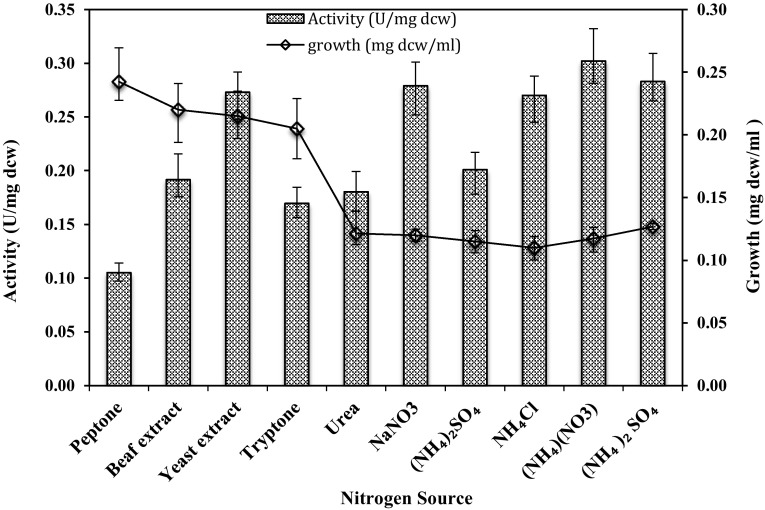
Amidase production by *G. subterraneus* RL-2a using various 3.0 % (w/v) nitrogen sources

To investigate the effect of various organic nitrogen sources on the production of amidase by *G. subterraneus* RL-2a, different organic nitrogen sources such as peptone, beef extract, yeast extract and tryptone at 0.3 % (w/v) were evaluated. Similarly, the effect of various inorganic nitrogen sources on the production of amidase by *G. subterraneus* RL-2a was evaluated. In general, organic nitrogen sources being more complex favoured more cell mass production compared to inorganic nitrogen sources (Fig. 2). The inorganic nitrogen sources repressed the cell mass production compared to organic sources. Yeast extract supported higher specific enzyme activity (0.29 U/mg dcw) and was chosen as nitrogen source in the following experiments.

### Taguchi experimental design for medium optimization for enhanced amidase production by *G. subterraneus* RL-2a

Any biological system is a highly complex process and the product formation depends upon the interaction of several physiological and environmental factors (agitation, medium pH, incubation temperature, time of incubation, etc.).

The interaction among environmental factors has enormous impact on cellular metabolism and subsequent product/enzyme production. Several combinations of environmental parameters and medium components were designed to obtain the best combination for the production of amidase from *G. subterraneus* RL-2a. The physical factors considered were medium pH, incubation temperature, and medium components were sucrose, K_2_HPO_4_, MgSO_4_·7H_2_O, NaCl, CaCl_2_·2H_2_O and yeast extract (Table 2).

Taguchi experimental design has been proved by various researchers as a good positive choice for the optimization of biotechnological processes for production of microbial enzymes over conventional methods. In this study, the eight factors were thought to have a significant effect on the amidase production. The results of the experiments, designed by the matrix, are illustrated in Table 3. The effects of the factors along with interactions at the assigned levels on the amidase production by *G. subterraneus* RL-2a are shown in Table 4, where yeast and NaCl showed the highest effect at level 1 and temperature, pH, sucrose, K_2_HPO_4_, MgSO_4_·7H_2_O and CaCl_2_·2H_2_O showed the highest effect at level 2.

**Table 4:** Estimated interaction of severity index for different factors

S. No.	Interacting factor pairs (order based on SI)	Columns	SI (%)	Reserved column	Levels
1	Temperature × NaCl	2 × 5	70.82	7	[2,1]
2	pH × sucrose	1 × 3	61.81	2	[2,1]
3	pH × MgSO_4_	1 × 7	61.43	6	[1,2]
4	K_2_HPO_4_ × CaCl_2_	4 × 8	61.06	12	[2,2]
5	K_2_HPO_4_ × NaCl	4 × 5	51.41	1	[3,1]
6	MgSO_4_ × CaCl_2_	7 × 8	47.68	15	[2,2]
7	Sucrose × CaCl_2_	3 × 8	41.61	11	[2,2]
8	Sucrose × MgSO_4_	3 × 7	41.49	4	[3,2]
9	Temperature × CaCl_2_	2 × 8	40.79	4	[2,1]
10	Temperature × sucrose	2 × 3	37.29	1	[2,3]
11	Temperature × yeast	2 × 6	32.12	4	[2,1]
12	Sucrose × yeast	3 × 6	31.81	5	[3,1]
13	Temperature × MgSO_4_	2 × 7	30.64	5	[1,1]
14	Yeast × CaCl_2_	6 × 8	29.11	14	[1,2]
15	Sucrose × NaCl	3 × 5	27.78	6	[3,1]
16	Yeast × MgSO_4_	6 × 7	25.5	1	[1,2]
17	pH × K_2_HPO_4_	1 × 4	25.42	5	[2,2]
18	NaCl × yeast	5 × 6	17.32	3	[1,1]
19	NaCl × CaCl_2_	5 × 8	14.6	13	[1,2]
20	pH × CaCl_2_	1 × 8	13.93	9	[1,2]
21	Temperature × K_2_HPO_4_	2 × 4	12.46	6	[2,3]
22	pH × yeast	1 × 6	12.24	7	[1,1]
23	K_2_HPO_4_ × yeast	4 × 6	10.97	2	[2,1]
24	Sucrose × K_2_HPO_4_	3 × 4	8.75	7	[3,3]
25	NaCl × MgSO_4_	5 × 7	7.31	2	[1,2]
26	pH × NaCl	1 × 5	3.63	4	[1,1]
27	pH × temperature	1 × 2	3.17	3	[2,2]
28	K_2_HPO_4_ × MgSO_4_	4 × 7	1.75	3	[3,2]

The larger the difference, the stronger the influence; the sign of the difference (+ or −) indicates whether the change from level 1 to level 2 or 3 increased or decreased the result. Based on these data it can be seen that K_2_HPO_4_, MgSO_4_·7H_2_O, and CaCl_2_·2H_2_O showed a stronger influence than that of other factors and the least influence was noticed with temperature and sucrose of the medium with the assigned levels (Table 1S).

**Fig. 3:**
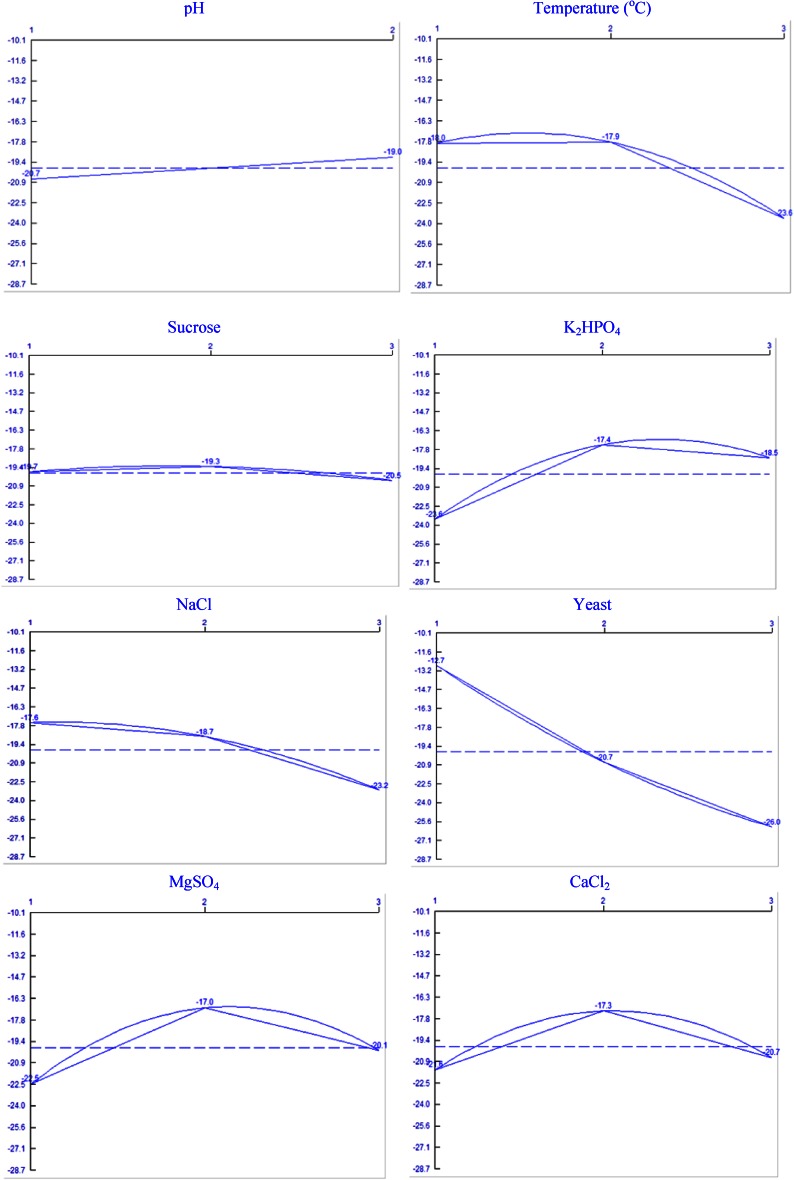
Performance of individual factors at different levels in relation to amidase production by *G. subterraneus* RL-2a

Increase in concentration of factors such as sucrose, K_2_HPO_4_ and increase in temperature and pH has resulted in increase in enzyme production up to level 2 and subsequent increase resulted in decrease in the amidase yield. Decrease in yeast extract and NaCl concentration has resulted in higher amidase expression up to level 1. While in case of MgSO_4_·7H_2_O and CaCl_2_·2H_2_O the amidase yield was higher at level 2, subsequent increase in the concentration to level 3 resulted in decrease in amidase yield. The influence of each individual factor on the amidase yield is shown in Fig. 3.

Any individual factor may interact with any or all of the other factors creating the possibility of presence of a large number of interactions. This kind of interaction is possible in Taguchi DOE.

The severity index (SI) was evaluated from Taguchi DOE that represents the influence of two individual factors at various levels of interaction (Table 4). In this table, the ‘columns’ represents the locations to which the interacting factors are assigned. Interaction SI presents 100 % of SI for 90° angle between the lines (factors), while 0 % SI for parallel lines. If the interaction between the factors is reverse, that can be shown by ‘reserved column’. ‘Levels’ indicated the level of factors desirable for the optimum conditions.

When interactions of different factors were calculated (Table 4), the highest interaction of SI (70.82 %) was observed in between Temperature and NaCl. It is interesting to note that the most little important factors such as pH versus sucrose (at their individual levels) interaction showed 61.81 severity index percentage. Similarly, the severity index percentage for sucrose (the least impact factor) versus yeast (the strong impact factor) was only 31.81 %. These results suggest that the influence of one factor on amidase production was dependent on the condition of the other factors in optimizing amidase production process parameters.

The variation of amidase production at chosen levels of each factor is depicted in Fig. 1S. Individually, each factor influenced amidase production at certain level. However, in combination, the yield was relatively low compared to optimized yield, which may be reasoned due to the interactive effect of different factors. The contribution of individual factor is the key to any fermentation process.

The percentage contribution of each factor is shown in an ANOVA (Table 5). The last column of the ANOVA indicates the influence of each factor. From the calculated ratios (*F*) it can be referred that all factors and interactions considered in the experimental design are statistically significant with 90 % of confident limit. Yeast extract was the most significant factor for amidase production. The next significant factors for the enzyme production in order of importance were temperature > K_2_HPO_4_ > NaCl > MgSO_4_·7H_2_O > CaCl_2_·2H_2_O > sucrose > pH.

**Table 5:** Analysis of variance (ANOVA)

S. No.	Factor	DOF (f)	Sums of sqrs (*S*)	Variance (*V*)	F-ratio (*F*)	Pure sum (*S*)	Percentage, *P* (%)
1	pH	1	12.25	12.25	14.92	11.43	1.06
2	Temperature	2	130.43	65.21	79.43	128.78	12.0
3	Sucrose	2	4.12	2.06	2.50	2.48	1.23
4	K_2_HPO_4_	2	128.05	64.02	77.99	126.41	11.78
5	NaCl	2	105.44	52.72	64.21	103.79	9.64
6	Yeast	2	537.68	268.84	327.47	536.04	49.96
7	MgSO_4_	2	91.96	45.98	56.01	90.32	8.42
8	CaCl_2_	2	61.31	30.65	37.34	59.67	5.56
Other/error		2	1.64	0.82			1.30
Total		17	1072.87				100.00

The confidence level for the temperature and NaCl were observed to be 95.37 and 98.4 %, respectively, while yeast extract was significant with a confidence level of 98.84 %. The remaining factors were significant at a confidence level below 95 %. K_2_HPO_4_ and pH showed negligible influence on the yield of amidase production at their individual levels.

Optimum condition of each factor and their performance in terms of contribution for achieving higher amidase yield was shown in Table 2S. It can be seen from the table that yeast extract has very significant role in the enzyme production than the other selected factors.

The contribution of individual factors is the key factors for the efficiency of fermentation process. The higher levels of amidase activity can be achieved with obtained optimization culture conditions: temperature: 55 °C pH 7.0; sucrose 10 g/l; K_2_HPO_4_ 2.5 g/l, yeast extract 0.1 g/l; NaCl 2.5 g/l; MgSO_4_·7H_2_O, 0.25 g/l; CaCl_2_·2H_2_O 0.5 g/l. It is evident from the Table 2S that upon considering the optimum culture condition from the designed experiments, the amidase yield can be increased from 0.29 to 0.65 U/mg dcw (Predicted value by Qualitek-4 software), i.e. overall 124.14 % increase in enzyme production can be achieved. To validate the proposed experimental methodology, production experiments were conducted by applying the obtained optimized culture condition as per the Table 2S. The obtained results confirmed an enhanced amidase yield of 0.62 U/mg dcw from 0.29 U/mg dcw (113.79 % increased in amidase yield) with the Taguchi DOE optimized culture condition.

After this statistical optimization the bacterium was again grown according to these physical and cultural parameters and the amidase yield as well as its growth was again assessed. The growth and enzyme production were found to increase according to the prediction given in the Taguchi DOE. Enzyme production following OVAT was about 0.29 U/mg dcw, but following the prediction of the Taguchi DOE it was about 0.62 U/mg dcw. So the increase in production was 113.79 % compared to initial amidase production.

While at the beginning of the experiments the production of amidase was about 0.23 U/mg dcw, after primary optimization of the culture conditions it was raised to 0.29 U/mg dcw (26.09 % increase). Using Taguchi optimization process, the enzyme produced was increased to 0.62 U/mg dcw, indicating a further increase of about (113.79 %) in production of amidase. As a result, enzyme production was finally increased to about 169.56 %, in relation to the initial step.

**Fig. 4:**
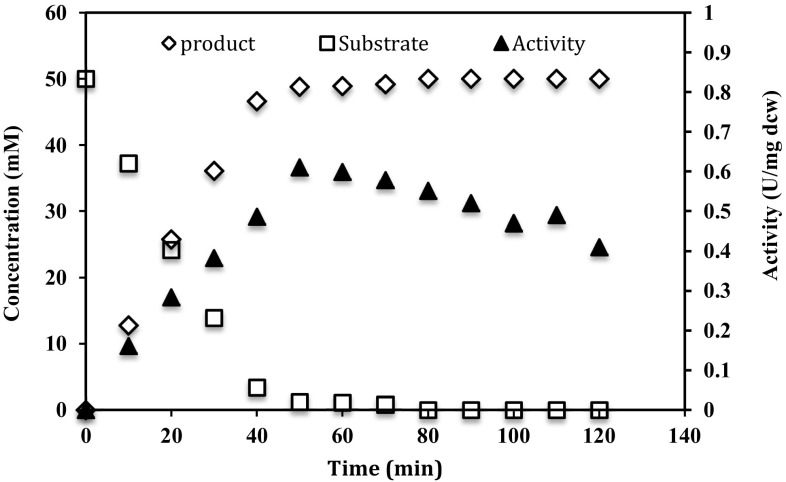
Effect of time duration on the amidase activity of *G. subterraneus* RL-2a and bioconversion of isonicotinamide to isonicotinic acid

The conversion time course of isonicotinamide by cells of *G. subterraneus* RL-2a with optimized medium is shown in Fig. 4. The conversion of isonicotinamide to isonicotinic acid was followed up to 120 min under the optimized medium conditions using 5.0 mg dcw of *G. subterraneus* RL-2a cells/ml. The maximum amidase activity was 0.59 ± 0.01 U/mg dcw after 50 min with 97 % conversion of isonicotinamide to isonicotinic acid (Fig. 4). Various studies have been reported on optimization of amidase production in bacterial strains using RSM (Vaidya et al. 2009; Wang et al. 2008). However, to the best of our knowledge, no reports are available on amidase production using Taguchi Methodology.

## Conclusion

To the best of our knowledge, there is not enough information concerning optimum nutritional requirements for amidase production by *G. subterraneus*. Using the one-factor-at-a-time and orthogonal array methods, it was possible to optimize nutritional components of medium to achieve higher amidase activity by *G. subterraneus* RL-2a. The methodology also facilitated understanding the specific functional role of eight factors involved in the amidase production using *G. subterraneus* RL-2a. Among the eight factors, yeast extract individually showed significant influence on maximum enzyme production. The amidase activity was improved with 2.7-fold initial activity under the optimized conditions and the conversion of isonicotinamide was significantly improved, which indicates suitability of this method in microbiological processes optimizations.

## Electronic supplementary material

Below is the link to the electronic supplementary material.

Supplementary material 1 (DOCX 18 kb)
