# Supplementary material for: Enhanced production of thermostable amidase from Geobacillus subterraneus RL-2a MTCC 11502 via optimization of physicochemical parameters using Taguchi DOE methodology
Source: 3 Biotech. 2016 Feb 15;6(1):66. doi: 10.1007/s13205-016-0390-1 (PMC4754296; doi:10.1007/s13205-016-0390-1)
Supplement: Supplementary file 1 — Supplementary material 1 (DOCX 18 kb) [file 13205_2016_390_MOESM1_ESM.docx]

**Fig. 1S**.

**Fig. 1S**. Relative influence of factors and interaction

**Table 1S.**

| Sr.No. | Factor | Level-1 | Level-2 | Level-3 | L_2_-L_1_ |
| --- | --- | --- | --- | --- | --- |
| 1 | pH | -20.66 | -19.01 | - | 1.649 |
| 2 | Temperature (°C) | -17.98 | -17.88 | -23.64 | 0.96 |
| 3 | Sucrose | -19.74 | -19.29 | -20.46 | 0.44 |
| 4 | K_2_HPO_4_ | -23.55 | -17.43 | -18.51 | 6.12 |
| 5 | NaCl | -17.58 | -18.73 | -23.19 | -1.15 |
| 6 | Yeast | -12.74 | -20.72 | -26.04 | -7.98 |
| 7 | MgSO_4._7H_2_O | -22.48 | -16.96 | -20.05 | 5.52 |
| 8 | CaCl_2._2H_2_O | -21.55 | -17.27 | -20.67 | 4.28 |

**Table 2S.**

| Sr.No. | Factor | Level description | Level | contribution |
| --- | --- | --- | --- | --- |
| 1. | pH | 7 | 2 | 0.82 |
| 2. | Temperature (°C) | 55 | 2 | 1.95 |
| 3. | Sucrose (g %, w/v) | 1 | 2 | 0.54 |
| 4. | K_2_HPO_4_ (g %, w/v) | 0.25 | 2 | 2.40 |
| 5. | NaCl (g %, w/v) | 0.25 | 1 | 2.25 |
| 6. | Yeast (g %, w/v) | 0.01 | 1 | 7.09 |
| 7. | MgSO_4_ (g %, w/v) | 0.025 | 2 | 2.87 |
| 8. | CaCl_2_ (g %, w/v) | 0.05 | 2 | 2.56 |
| Total contribution from all factors 20.49  Current Grand average of performance -19.83  Expected result at optimum condition 0.65 | | | | |

**Table 1S.** Main effects of selected factors

**Table 2S.** Optimum of culture conditions and their contribution
